# Differences in rhizospheric microbial communities between cultivated and wild endangered *Glyptostrobus pensilis*

**DOI:** 10.3389/fmicb.2025.1548836

**Published:** 2025-03-27

**Authors:** Xiaojuan Yang, Biyu Deng, Shiyi Lu, Cong Wang, Yongyan Liang, Shinan Liu

**Affiliations:** ^1^Guangxi Key Laboratory of Forest Ecology and Conservation, College of Forestry, Guangxi University, Nanning, China; ^2^Guangxi Forest Resources and Environment Monitoring Center, Nanning, China; ^3^Guangxi Beihai Wetland Ecosystem National Observation and Research Station, Beihai, China

**Keywords:** *Glyptostrobus pensilis*, endangered tree species, root, soil, microbial communities, soil physicochemical properties

## Abstract

*Glyptostrobus pensilis* is an endangered species belonging to the Cupressaceae family. The comprehensive examination of soil characteristics and rhizosphere microbial communities is vital for conservation efforts, as it provides insights into the necessary environmental conditions for safeguarding and ensuring the viability of rare and endangered species. In this study, the diversity and composition of bacterial and fungal communities were compared in the roots and rhizosphere soils of cultivated and wild *G. pensilis* in Guangxi, China. The results revealed that, at the phylum level, the rhizosphere of cultivated *G. pensilis* was significantly enriched with *Verrucomicrobiota*, *Acidobacteriota*, *Glomeromycota*, and *Chloroflexi*, while wild *G. pensilis* was significantly enriched with *Planctomycetota*, *Basidiomycota*, and *Ascomycota*. Symbiotic network analysis indicated that the bacterial network in the cultivated *G. pensilis* rhizosphere had higher edge values, average degree, clustering coefficient, and network density, while the fungal network in the wild *G. pensilis* rhizosphere had higher node values, edge values, average degree, and clustering coefficient. Moreover, functional prediction results suggested that bacteria in cultivated *G. pensilis* showed higher metabolic activity, with fungi primarily acted as saprotrophs and symbionts. In contrast, bacteria in wild *G. pensilis* displayed lower metabolic activity, with fungi predominantly functioning as saprotrophs. The analysis linking rhizospheric microbial diversity to soil environmental factors showed a closer association for the wild *G. pensilis* microbial community, suggesting a stronger influence of soil environmental factors. The Random Forest (RF) highlighted that the total phosphorus and total potassium levels were key influencing factors for rhizospheric microbes in cultivated *G. pensilis*, while available potassium levels were crucial for those in wild *G. pensilis*. These differences underscore the significant strategies for *G. pensilis* in adapting to different habitats, which may be intricately linked to land management practices and soil environmental factors. Among these, phosphorus and potassium are significantly associated with the rhizosphere microorganisms of *G. pensilis*. Therefore, continuous monitoring of nutrient availability and regular supplementation of phosphorus and potassium fertilizers in the rhizosphere are recommended during the cultivation and ex-situ conservation of *G. pensilis*.

## 1 Introduction

The rhizosphere is the primary region of the interaction between plant roots and soil, which hosts a vast array of plant-associated microbial communities including bacteria and fungi (Park et al., 2023). Plant roots can selectively recruit specific rhizospheric microorganisms to alleviate abiotic stress, enhance plant resistance, and facilitate nutrient cycling and metabolism (Deng et al., 2024; Parasar et al., 2024; You et al., 2024). These microbial communities are influenced by root exudates and environmental factors, which shape soil elemental cycles and affect plant performance (Dhungana et al., 2023). For example, tomato (*Solanum lycopersicum*) roots secrete plant secondary metabolites (PSMs) to attract beneficial microorganisms that assist in nutrient availability and uptake (Nakayasu et al., 2023). Furthermore, under the influence of root exudates from sugar beet (*Beta vulgaris*), the diversity of rhizospheric microoranisms becomes more complex, promoting the accumulation of soil inorganic nitrogen (Liu S. L. et al., 2024). Therefore, it is of significance to investigate the correlations between microorganisms and plants.

Recently, studies have emphasized the significant role of rhizospheric microbial communities in influencing endangered plant nutrient uptake and environmental adaptation. Firstly, arbuscular mycorrhizal fungi (AMF) can promote the growth and nutrient acquisition of endangered plants. For example, *Glomus constrictum* and *Glomus versiforme* promote the growth, nutrient absorption, and accumulation of medicinal components in the endangered medicinal plant *Fritillaria* L. (Zhou et al., 2022). In the endangered tree species *Heptacodium miconioides*, inoculation with *Rhizophagus intraradices* can significantly alleviate the harmful effects of drought stress (Li et al., 2023). Additionally, inoculation with *Glomus coronatum* improves the survival of the endangered plant *Kalappia celebica* by accelerating its growth (Husna et al., 2021). Secondly, the presence or absence of specific microbial communities influences the adaptability of endangered plants to their environment. For instance, *Udaeobacter* spp. enhances the adaptability of *Firmiana danxiaensis* in soils characterized by a high nitrogen-to-phosphorus ratio and phosphorus deficiency (Li et al., 2025). Conversely, the absence of beneficial strains such as *Suillus*, *Geomyces*, and *Gigaspora* maycontributed to the slow growth and development of the endangered plant *Ostrya rehderiana* Chun (Tong et al., 2021). Furthermore, the composition of rhizosphere fungal communities can serve as an indicator of ecosystem health and play a crucial role in the restoration and conservation of habitats for endangered plants. For example, fungal communities such as *Archaeorhizomyces*, *Dactylella*, and *Helotiales* are recognized as important indicators of habitat health for the endangered plant *Scutellaria tsinyunensis* (Zuo et al., 2022b).

*Glyptostrobus pensilis* (Staunton ex D. Don) K. Koch, a relict species in the family Cupressaceae, is listed as critically endangered on the Red List of the International Union for Conservation of Nature (IUCN). Its wood is characterized by the lightweight and soft texture, coupled with a high resistance to decay. Extracts exhibit anti-inflammatory, antibacterial, and anticancer activities, with certain compounds potential for the treatment of chronic myeloid leukemia and COVID-19 (Xiong et al., 2020; Phong et al., 2022; Zhu et al., 2024), highlighting its economic and medicinal significance. Furthermore, *G. pensilis* possesses a well-developed root system that enables it to withstand heavy metal pollution, particularly from chromium (Cr) and nickel (Ni). This capability also contributes to its role in soil and water conservation and supports carbon neutrality (Ye et al., 2022). Fossil records suggest that *G. pensilis* was once widely distributed across the Northern Hemisphere; however, due to climate change and human activities, its current populations are restricted to central Vietnam, eastern Laos, and southern China (Tang et al., 2019; Zhang Y. Z. et al., 2024). In China, *G. pensilis* primarily thrives wetlands located in Fujian, Guangxi, and Guangdong, yet wild individuals remain exceedingly rare (Pueyo-Herrera et al., 2023).

Previous studies on *G. pensilis* have primarily concentrated on its physiological, biochemical, genetic, and embryological aspects (Huy Thai et al., 2023; Wu et al., 2020; Yuan et al., 2023). In the realm of microbiological research, Zhang C. et al. (2024) conducted the first analysis of seasonal variations in the rhizosphere microorganisms associated with *G. pensilis*, revealing that microbial diversity and carbon metabolism capacity peaked during the summer and significantly declined in winter. However, the differences in rhizosphere microorganisms of *G. pensilis* across various habitats remain unclear. This study aims to investigate the composition, diversity, and differences in rhizospheric microbial communities between wild and cultivated *G. pensilis*, elucidate their symbiotic networks and functional characteristics, and identify the influencing factors. The findings will provide a scientific foundation for understanding the microbiome of *G. pensilis*and offer valuable resources for habitat restoration and conservation efforts.

## 2 Materials and methods

### 2.1 Sample collection

According to our preliminary survey, only seven wild *G. pensilis* individuals remained in Guangxi, with one each being found in Qintang District, Cangwu County, Binyang County, Tiandeng County, Pingle County, and two in Yanshan District. Besides, a limited number of cultivated *G. pensilis* are primarily found in Tiandeng County and Pingle County. In July 2023, we collected samples from cultivated and wild *G. pensilis* across different regions in Guangxi, China. The cultivated samples were gathered at three sites from Tiangdeng and Pingle counties (Supplementary Table 1). At each site within the same habitat, three trees were randomly selected as replicate samples, ensuring a minimum distance of 5 m between individuals (Zuo et al., 2022a; Liu J. X. et al., 2024; Li F. R. et al., 2024). The wild samples were gathered at six sites from Tianteng County, Yanshan District, Cangwu County, Binyang County, Pingle County, and Qingtang District. Due to the solitary nature of wild *G. pensilis*, only one tree at each site except Yanshan were identified and sampled. Moreover, two trees were found at the Yanshan site, which were spaced less than five meters apart, and their roots or rhizosphere soil were combined to form a single sample. In total, 15 rhizosphere soil samples and 15 root samples were collected.

Our sampling methodology was executed as follows: initially, surface litter and fallen leaves were removed using a small shovel; next, roots were excavated from a depth of 0–20 cm around the plant. The loosely attached soil was shaken off, with portions reserved for enzyme activity analysis (kept at −20°C) and physicochemical property analysis (air-dried and stored at room temperature). In addition, rhizosphere soil within 1–10 mm of the root surface was collected, thoroughly mixed in sterile bags, and stored with root segments in sampling tubes. The collected samples were immediately flash-frozen in liquid nitrogen, transported to the laboratory on dry ice, and stored at −80°C for further analysis.

### 2.2 Soil physicochemical properties and enzyme activity determination

Soil physicochemical properties were determined following the methodology outlined by Bao (2000). Soil moisture content (SMC) was measured using the drying method, while pH was determined using the acidity titration method. Soil organic carbon (SOC) was measured using the potassium dichromate oxidation-oil bath heating method. Total nitrogen (TN) was analyzed using the semi-micro Kjeldahl method and total phosphorus (TP) was determined by the Mo-Sb colorimetric method. Total potassium (TK) was measured using the NaOH melt flame method. Soil ammonium nitrogen (AN) and nitrate nitrogen (NN) concentrations were determined by UV spectrophotometry. Available phosphorus (AP) was measured by the hydrochloric acid-sulfuric acid extraction method, and available potassium (AK) was assessed using the ammonium acetate method. Additionally, the activities of β-1,4-glucosidase (BG), β-D-glucosidase (CBH), β-1,4-N-acetylglucosaminidase (NAG), leucine aminopeptidase (LAP), and alkaline phosphatase (ALP) were measured using the microplate fluorometric method (Chen et al., 2021).

### 2.3 Rhizospheric microbial DNA extraction and high-throughput sequencing

The FastDNA Spin Kit for Soil (MP Biomedicals, Santa Ana, CA, USA) was used to extract total DNA from rhizosphere soil and root-associated microorganisms. The purity, concentration, and quality of the DNA (A260/280 ratio) were measured using a NanoDrop 2000 spectrophotometer (Thermo Fisher Scientific, Wilmington, DE, USA), and its integrity was assessed via 1% agarose gel electrophoresis. The V5-V7 region of the 16S rRNA gene and the ITS1 region of rhizosphere microbial DNA were amplified using the specific primers 799F (AACMGGATTAGATACCCKG)/1193R (ACGTCATCCCCACCTTCC) and ITS1F (CTTGGTCATTTAGAGGAAGTAA)/ITS2 (GCTGCGTTCTTCATCGATGC), respectively, following the methods described by Li et al. (2021) and He et al. (2022). After purifying the amplicons using standard protocols, paired-end sequencing was performed on an Illumina NovaSeq 6000 platform (Illumina Inc., San Diego, CA, USA).

### 2.4 Bioinformatics and statistical analyses

The sample data were demultiplexed based on barcode and PCR primer sequences. Paired-end reads were merged using FLASH (version 1.2.11), primer sequences were trimmed with Cutadapt, and data quality control was performed using FASTP (version 0.23.1). Chimeric sequences were removed using a reference database to obtain high-quality effective data. The resulting Effective Tags were denoised using the DADA2 module in QIIME2 (v202202) to generate the final ASVs (Amplicon Sequence Variants) and feature table.

To compare the differences between wild and cultivated *G. pensilis*, we made the following comparisons of their rhizospheric bacteria and fungi: wild rhizosphere soil vs. cultivated rhizosphere soil (WS vs. CS), and wild roots vs. cultivated roots (WR vs. CR). Meanwhile, to reduce the impact of the experimental specificity on the results, we performed correlation analysis between microorganisms and soil environmental factors for each habitat using single replicate samples, summarized the common patterns of each habitat, and made comparisons based on these. The specific analysis is as follows: a species-level phylogenetic tree was constructed using the top 100 abundant genera to compare the differences in microbial communities. In the LEfSe analysis, the Kruskal-Wallis test was used to determine the significance of differences, and LDA analysis was applied to assess the impact of these differences on sample grouping. Significant differential biomarkers with a classification score > 4.0 between groups were identified. Furthermore, the abundance and diversity indices (Chao1, Shannon, and Simpson) of each taxon were analyzed using MOTHUR (version 1.31.2). Afterwards, unweighted pair-group method with arithmetic means (UPGMA) clustering analysis was conducted on the basis of the Weighted Unifrac distance matrix to assess the similarity between community structures (beta diversity). To characterize the differences in soil microbial symbiotic patterns, the Spearman correlation matrix was calculated among the top 100 abundant species. Strong correlations with an average relative abundance greater than 0.00005 (*R* > 0.6, *p* < 0.05) were retained and visualized with Gephi (version 0.10.1), and the top five genera were determined as key taxa based on the intermediary centrality scores (Gao and Zhang, 2023).

## 3 Results

### 3.1 Rhizospheric bacterial and fungal community compositions

**Figure 1:**
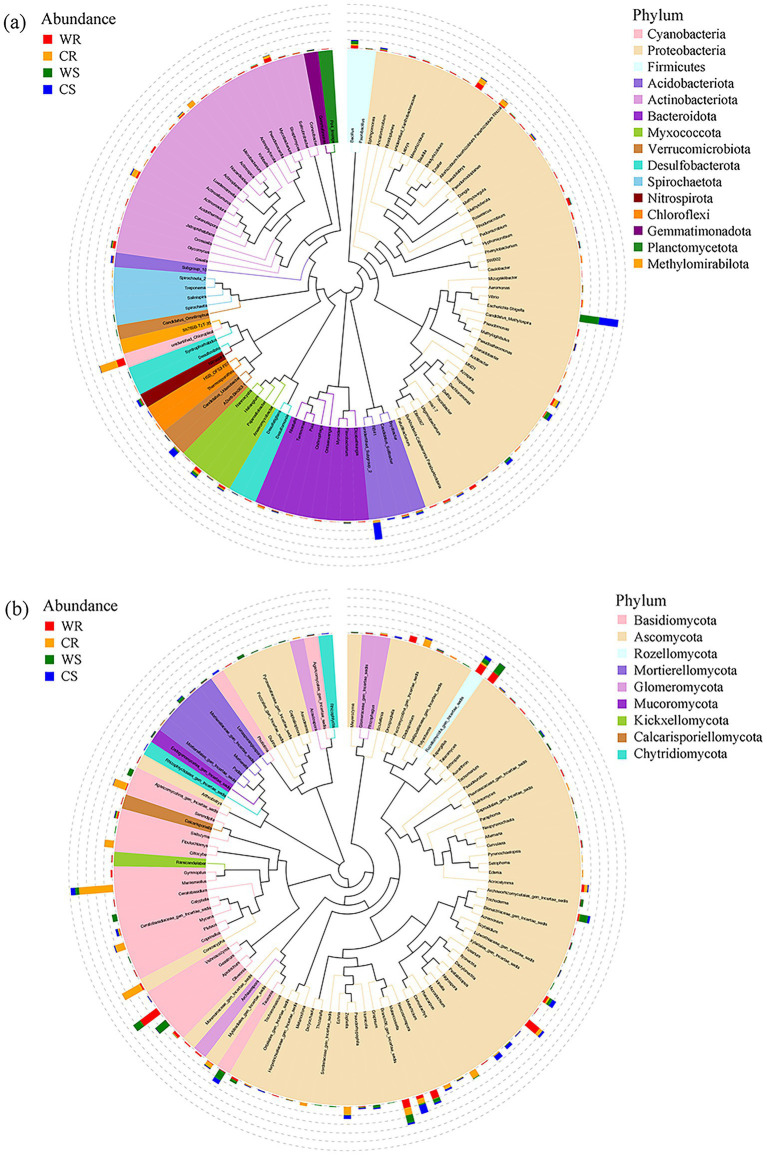
The phylogenetic trees constructed for the top 100 bacterial **(a)** and fungal **(b)** genera. The colors of branches and sectors indicate their corresponding phyla. The stacked bar plots outside the sector ring depict the abundance distribution information of each genus in cultivated *G. pensilis* root (CR), wild *G. pensilis* root (WR), cultivated *G. pensilis* soil (CS) and wild *G. pensilis* soil (WS).

In bacterial communities, the wild *G. pensilis* root (WR), cultivated *G. pensilis* root (CR), wild *G. pensilis* soil (WS), and cultivated *G. pensilis* soil (CS) obtained totally 24,956, 22,685, 27,429, and 22,052 ASVs, respectively. These ASVs belong to 432, 412, 464, and 421 phyla and 812, 729, 790, and 719 genera. In fungal communities, WR, CR, WS, and CS obtained 2,777, 2,417, 5,317, and 4,821 ASVs, respectively. These ASVs belong to 231, 223, 317, and 306 phyla and 406, 383, 618, and 573 genera. The phylogenetic trees showed the top 100 abundant bacterial genus and top 100 abundant fungal genus, encompassing 15 bacterial phyla and nine fungal phyla (Figure 1).

The majority of bacterial genera belonged to *Proteobacteria* (43 genera), *Actinobacteriota* (20 genera), *Bacteroidota* (8 genera), *Acidobacteriota* (5 genera), *Myxococcota* (4 genera), *Desulfobacterota* (4 genera), and *Spirochaetota* (4 genera), while the remaining belonged to *Verrucomicrobiota*, *Cyanobacteria*, *Firmicutes*, *Nitrospirota*, *Chloroflexi*, *Gemmatimonadota*, *Planctomycetota*, and *Methylomirabilota* (Figure 1a). Most of the fungal genera were in *Ascomycota* (65 genera), *Basidiomycota* (20 genera), *Mortierellomycota* (5 genera), and *Glomeromycota* (4 genera), whereas the rest belonged to *Chytridiomycota*, *Rozellomycota*, *Mucoromycota*, *Kickxellomycota*, and *Calcarisporiellomycota* (Figure 1b).

Notably, the top 7 bacterial genera included unidentified_Chloroplast, *Escherichia-Shigella*, *Uliginosibacterium*, *Bacillus*, *Allorhizobium-Neorhizobium-Pararhizobium-Rhizobium*, unidentified_Subgroup_2, and *Actinoallomurus*, which collectively occupied around 30% of the bacterial communities (Figure 1a); the top 5 fungal genera were *Marasmiellus*, *Conioscypha*, *Geastrum*, *Clitocybe*, and *Dactylonectria*, accounting for approximately 22% of the fungal communities (Figure 1b).

Regarding bacterial microbiota, in cultivated *G. pensilis* root (CR) and wild *G. pensilis* root (WR), *Paludibacterium*, *Elizabethkingia*, *Glycomyces*, *Mizugakiibacter*, *Myroides*, and *Methylovirgula* were exclusively found in WR, while *Thermosporothrix* was exclusively detected in CR (Figure 1a). In cultivated *G. pensilis* soil (CS) and wild *G. pensilis* soil (WS), *Actinoallomurus*, *Vibrio*, *Myroides*, and *Methyloferula* were exclusively discovered in WS, while *Catenulispora* and *Ancalomicrobium* were exclusively detected in CS (Figure 1a). In terms of fungal microbiota, in CR and WR, seven genera were exclusively observed in CR, while 24 were exclusively detected in WR. In CS and WS, there were three genera exclusively observed in CS and 12 in WS, respectively (Figure 1b).

### 3.2 LEfSe analysis of rhizospheric microorganisms

**Figure 2:**
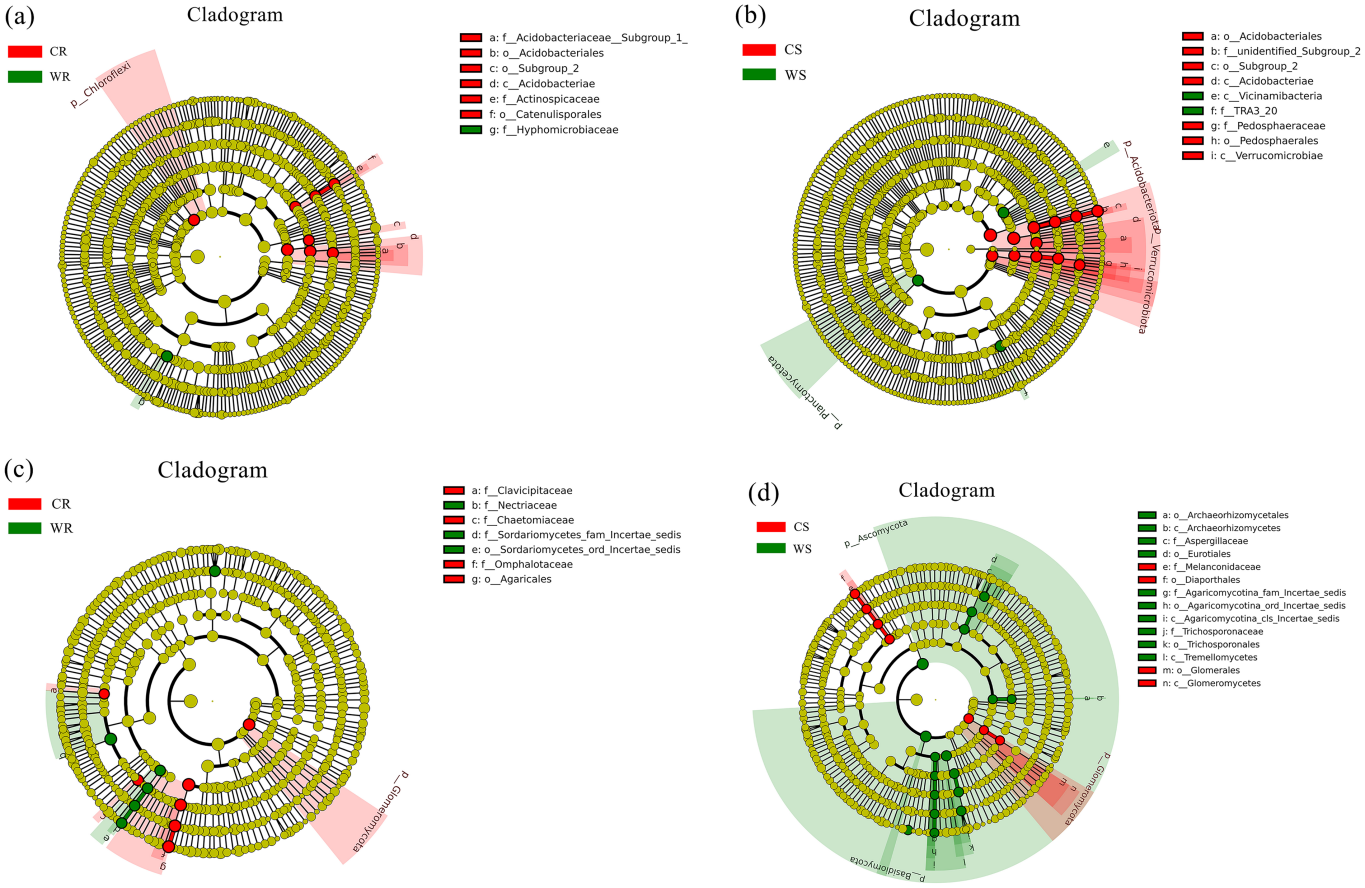
The LDA effect size (LEfSe) of bacterial **(a,b)** and fungal **(c,d)** communities, with LDA scores >4.0 (*p* < 0.05). The radiating circles from the inside out refer to the classification hierarchy from phylum to genus (or species). Each small circle at varying classification levels represents a category at that level, with the diameter of the small circle being proportional to the relative abundance. Species exhibiting no significant differences are uniformly colored yellow, while differentially abundant species are colored in accordance with the biomarker-followed group.

**Figure 3:**
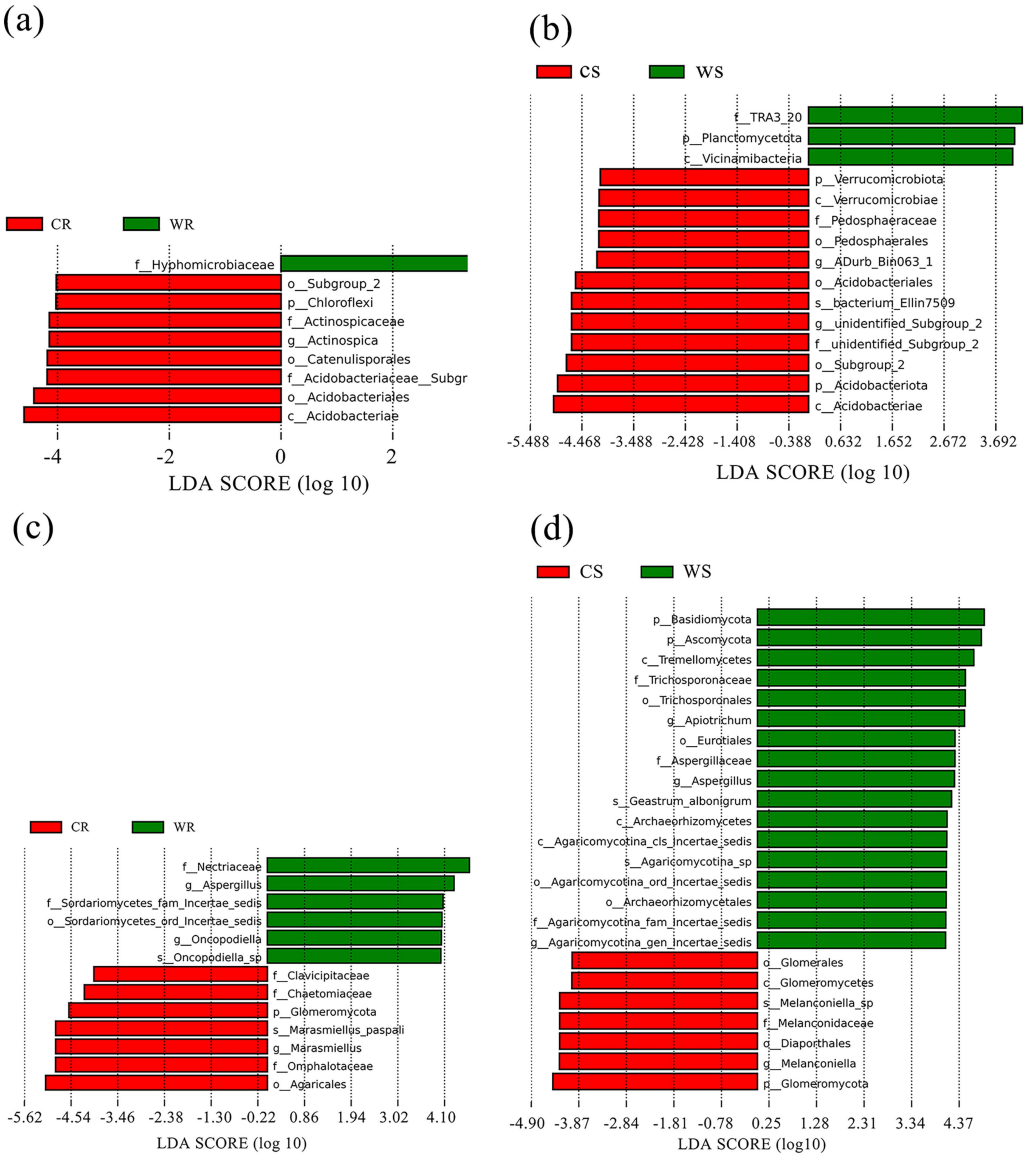
The LDA value distribution histograms of bacterial **(a,b)** and fungal **(c,d)** communities.

LEfSe analysis was used for identifying the significantly different microbiota between the cultivated and wild *G. pensilis*. In the CR, WR, CS, and WS, 8, 1, 12, and 3 differential bacterial communities (Figures 2a,b) and 7, 6, 7, and 17 differential fungal communities (Figures 2c,d) were identified, respectively. The biomarker number of bacterial and fungal in CR and CS was higher than in WR and WS, except for the fungal in CS. At the phylum level, *Chloroflexi* and *Glomeromycota* were significantly enriched in CR; while *Planctomycetota*, *Basidiomycota*, and *Ascomycota* were significantly enriched in WS; in addition, *Verrucomicrobiota*, *Acidobacteriota*, and *Glomeromycota* were significantly enriched in CS (Figure 3). *Acidobacteriae* and *Agaricales* with linear discriminant analysis (LDA) scores >5.0 exhibited highly significant differences in the bacterial and fungal communities of CS and CR, respectively (Figures 3b,c).

### 3.3 Diversity of rhizospheric microbial communities

**Figure 4:**
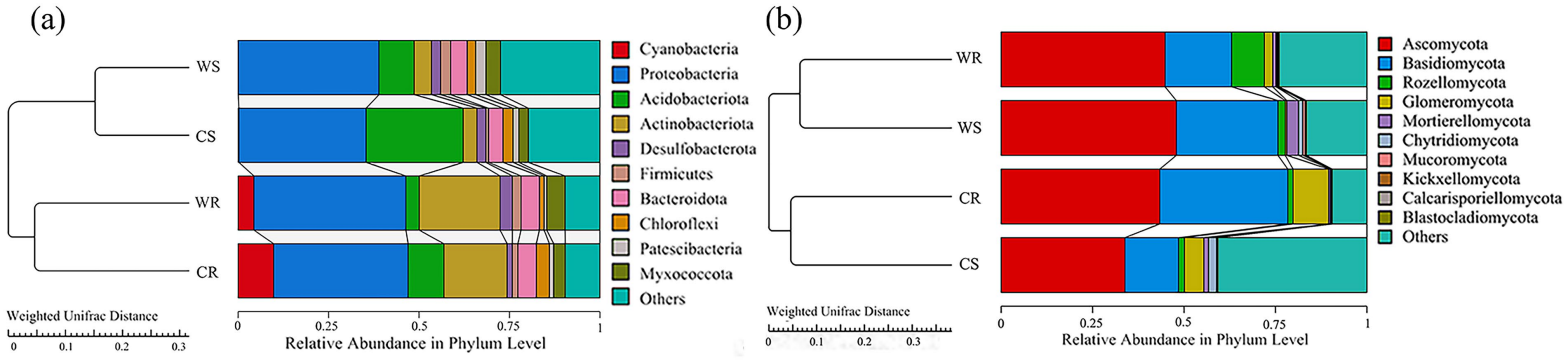
The UPGMA clustering trees of bacteria **(a)** and fungi **(b)** constructed on the basis of the Weighted Unifrac distance.

Analysis of beta diversity indicated that, in terms of bacteria, WR and CR clustered together, and that WS and CS clustered together (Figure 4a); with regard to fungi, WR and WS clustered together, while CR and CS clustered together (Figure 4b).

### 3.4 Symbiotic networks of bacterial and fungal communities

**Figure 5:**
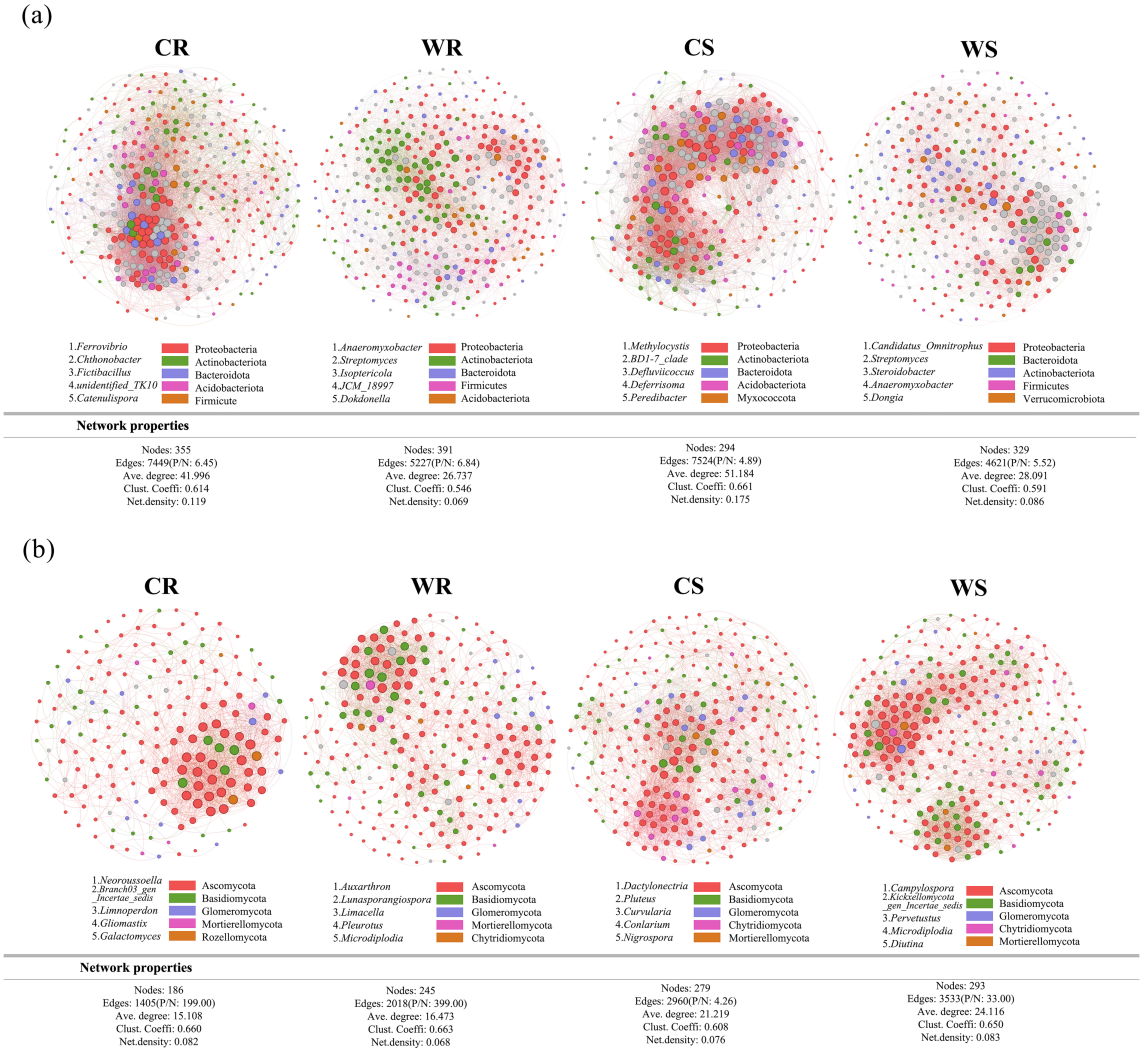
The symbiotic networks of core bacterial **(a)** and fungal **(b)** microbiota. Different nodes indicate different genera, with the node size representing the average relative abundance of the genus. Nodes of the same phylum are colored the same, the thickness of the lines between nodes exhibits a positive relationship to the absolute value of the species interaction coefficient, and the line color is consistent with the positive or negative correlation (red for positive, and green for negative). The annotated numbers represent key genera in the network (top five based on the betweenness centrality scores).

Based on symbiotic network analysis of microbiota (both bacteria and fungi), the positive/negative (P/N) ratios of bacteria and fungi in WR and WS were higher than those in CR and CS, respectively (Figure 5). In the bacterial network, the values of edges, average degree, clustering coefficient, and network density in CR and CS were higher than those in WR and WS, respectively (Figure 5a). By contrast, the fungal network exhibited lower values for nodes, edges, average degree, and clustering coefficient in CR and CS than WR and WS, respectively (Figure 5b). Therefore, the bacterial network of wild *G. pensilis* is simpler than that of cultivated *G. pensilis*, whereas the fungal network is more complex.

### 3.5 Functional composition profiles of microbial communities

KEGG pathways (level 2) were selected to predict the metabolic potential of bacterial microbiota. Our results indicated that the majority of bacterial metabolic pathways were mainly associated with membrane transport, amino acid metabolism, and carbohydrate metabolism (Supplementary Figure 1a). In CR, significantly enriched pathways included poorly characterized, glycan biosynthesis and metabolism, enzyme families, biosynthesis of other secondary metabolites, and signaling molecules and interaction (Supplementary Figure 1c). In WR, the significantly enriched pathways were metabolism of terpenoids and polyketides pathway (Supplementary Figure 1c). A comparison of rhizosphere soil microbiota revealed 17 significantly different functional guilds between wild and cultivated *G. pensilis*, with 11 significantly enriched in CS and 6 significantly enriched in WS (Supplementary Figure 1d). Evidently, the bacteria in cultivated *G. pensilis* exhibited higher metabolic activity. Using FUNGuild, the functional composition of fungal communities in *G. pensilis* roots and rhizosphere soil was predicted, exhibiting nine nutritional types, primarily saprotroph, symbiotroph, pathotroph-saprotroph, and pathotroph (Supplementary Figure 1b). Saprotroph occupied the largest proportion in WS, reaching 39.28%, significantly higher than that in CS (Supplementary Figures 1b,e,f). The symbiotic type in CR and CS was significantly higher than in WR and WS (Supplementary Figures 1e,f). Obviously, the saprotroph-pathotroph-symbiotroph type was only observed in the cultivated *G. pensilis*. Clearly, the fungi of cultivated *G. pensilis* function mainly as symbionts and saprotrophs, while those of wild *G. pensilis* primarily act as saprotrophs.

### 3.6 Correlation between microbial communities and environmental factors

The control of biological processes in *G. pensilis* varies among different habitats. According to the bacterial community function prediction results and their comparisons with KEGG (level 1), it was found that the functions involved in metabolism, genetic information processing, unclassified, environmental information processing, cellular processes and organismal systems (Supplementary Figures 2a,b). Furthermore, the alpha diversity index of rhizosphere microorganisms is presented in Supplementary Table 3. Based on Mantel analysis of rhizospheric microbiota and its environmental factors, fungal microbiota in the cultivated *G. pensilis* was less impacted by the soil environment, while bacterial diversity and functional structure were significantly correlated with AP, pH, NN, BG, CBH, NAG, and SMC (Supplementary Figure 2a). The fungal function in the wild *G. pensilis* was significantly related to TP, TK, AP, AK, BG, CBH, NAG, and LAP, while fungal diversity was significantly associated with SOC, TN, TP, pH, LAP, ALP, and SMC. The bacterial function was significantly correlated with TP, pH, LAP, and SMC, while bacterial diversity was pH, LAP, and SMC (Supplementary Figure 2b). Among them, fungal function was highly significantly related to TP, AP, and AK (*p* < 0.01), while fungal diversity was highly significantly correlated with LAP and SMC.

**Figure 6:**
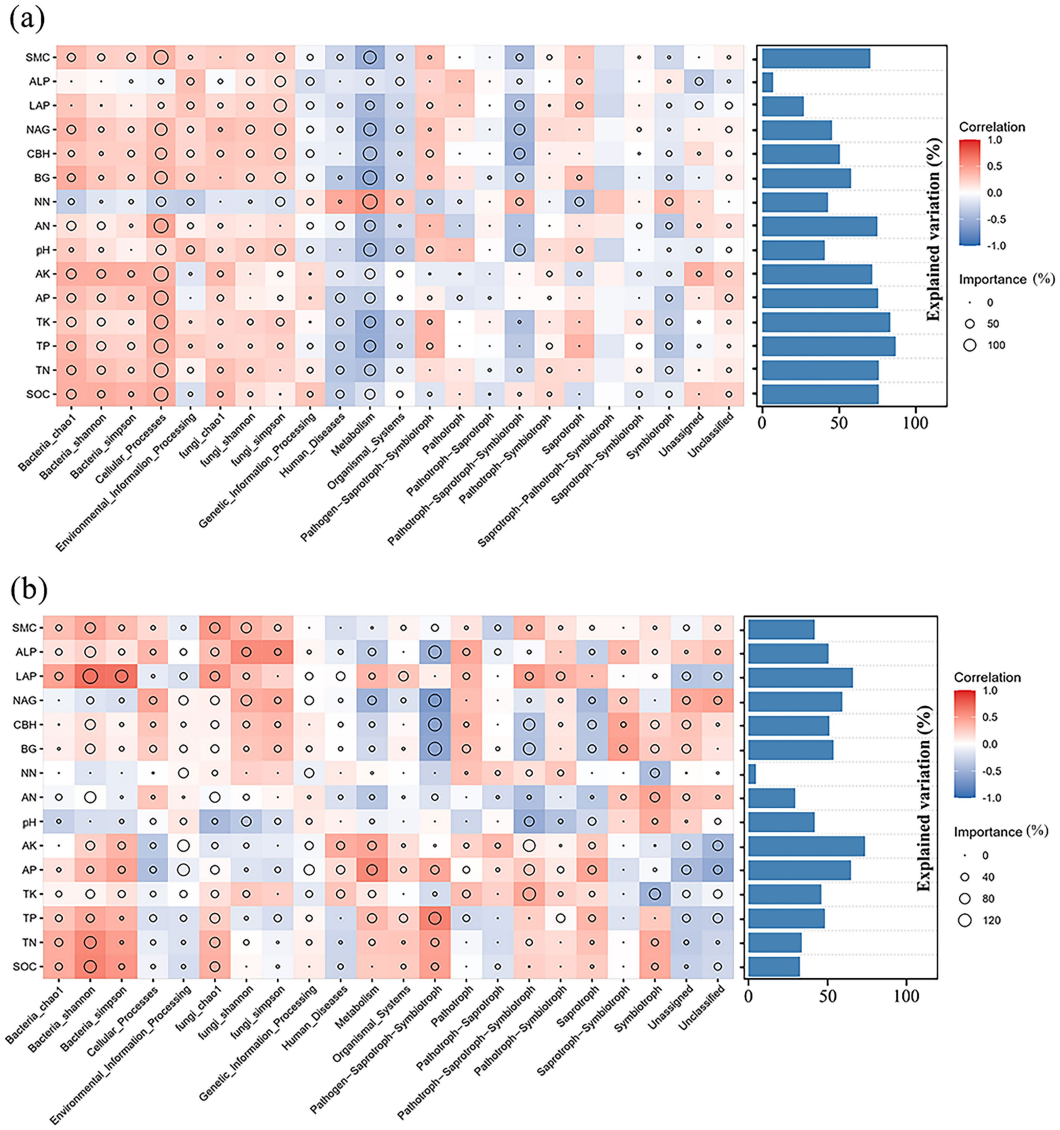
The potential contributions of environmental factors to the functional composition and Alpha diversity of microbial communities in the cultivated *G. pensilis* **(a)** and wild *G. pensilis* **(b)**. The bar chart exhibits the degree of explanation for the microbial community. The size of the circles (RF) indicates the importance of variables. The depth of the color represents the relative strength of Spearman correlation.

The biological relationship between microbial diversity in *G. pensilis* and environmental factors was assessed using the multivariate regression models and RF analysis. It was indicated that TP and TK exerted the greatest effect on the abundance and functional structure of microbial communities in the cultivated *G. pensilis*, followed by SMC, AN, AK, AP, TN and SOC, which were all positively correlated with rhizosphere microbial diversity (Figure 6a). AK was one of the most vital factors influencing the abundance and functional structure of microbial communities in the wild *G. pensilis*, followed by LAP, NAG and AP (Figure 6b). AK and AP were negatively related to fungal diversity and positively correlated with bacterial diversity. LAP and NAG were positively associated with both fungal and bacterial diversity.

## 4 Discussion

Differences exist in the composition and structure of plant rhizosphere microbial communities under different habitat conditions, which represent a crucial ecological strategy for environmental adaptation (Ni et al., 2023; Yuan et al., 2023). Research has demonstrated that forest management practices can affect the rhizosphere microorganisms of apple plants (*Malus pumila*) (Xie et al., 2022), Chinese fir (*Cunninghamia lanceolata*) (Lei et al., 2023), and *Amomum villosum* (Wang B. T. et al., 2023). Similarly, our study observed analogous phenomena.

In comparison to wild *G. pensilis*, the cultivated ones exhibited a significant enrichment of bacterial groups such as *Chloroflexi*, *Actinospicaceae*, and *Acidobacteriales* in their roots (Figure 3a), along with fungal groups including *Glomeromycota*, *Omphalotaceae*, and *Agaricales* (Figure 3c). Moreover, bacterial taxa including *Verrucomicrobiota*, *Acidobacteriota*, and *Acidobacteriae* (Figure 3b), as well as fungal groups like *Glomeromycota*, *Melanconidaceae*, and *Melanconiella* (Figure 3d), were notably enriched in the rhizosphere soil. Additionally, functional annotation T-test results revealed that endophytic bacteria in cultivated *G. pensilis* were remarkably enriched in pathways related to enzyme families, signaling molecules and interactions, and glycan biosynthesis and metabolism (Supplementary Figure 1c). In contrast, the rhizosphere soil bacteria were notably enriched in pathways associated with carbohydrate metabolism, enzyme families, transport and catabolism, and signaling processes (Supplementary Figure 1d). Furthermore, both the roots and rhizosphere soil showed a notable accumulation of symbiotrophic fungi (Supplementary Figures 1e,f). It has been established that *Chloroflexi*, *Acidobacteriota*, and *Verrucomicrobiota* can degrade complex carbon sources such as cellulose, hemicellulose, and lignin (Yao et al., 2024; Gonçalves et al., 2024; Rakitin et al., 2024), thereby promoting the release of available carbon (Li G. T. et al., 2024). Simultaneously, taxa associated with enzyme families accelerate organic matter mineralization by secreting degrading enzymes, thus increasing the availability of nitrogen and phosphorus (Liu D. L. et al., 2024; Zhang K. et al., 2024). Collectively, these mechanisms enhance the soil’s capacity to supply nutrients. Furthermore, Guo et al. (2020) and Narsing Rao et al. (2022) have noted that *Glomeromycota* and *Actinospicaceae* can facilitate nutrient uptake. The enrichment of symbiotic microorganisms contributes to the formation of stable networks, which optimize nutrient use efficiency (Guo et al., 2020). Such microbial interactions enhance the plant’s ability to acquire soil resources. Additionally, transport and catabolic pathways can boost microbial metabolic activity, while signaling molecules and interaction pathways can regulate microbial community structure through quorum sensing (QS) and phytohormone modulation (Kalia et al., 2021), thereby strengthening the plant’s adaptability to environmental stress. In summary, the microorganisms significantly enriched in cultivated *G. pensilis* play a critical role in soil nutrient supply, nutrient acquisition, and environmental adaptation. However, our study found that the overall soil nutrient content in cultivated *G. pensilis* under land management is lower than that in wild ones (Supplementary Table 2), a finding consistent with observations in *Quercus palustris* (Lei et al., 2023), *Cunninghamia lanceolata* (Zhou et al., 2020), and *Eucalyptus* (Mofini et al., 2022). Therefore, we hypothesize that the structure of the rhizosphere microbial community in cultivated *G. pensilis* represents an adaptive strategy to low-nutrient soil conditions.

The roots of wild *G. pensilis* were enriched with a specific bacterial group (*Hyphomicrobiaceae*) (Figure 3a) and multiple fungal groups (e.g., *Nectriaceae*, *Aspergillus*, and *Oncopodiella*) (Figure 3c). Notably, bacterial taxa such as *TRA3_20*, *Planctomycetota*, and *Vicinimibacteria* (Figure 3b), along with fungal taxa including *Basidiomycota*, *Ascomycota*, *Tremellomycetes, Eurotiales*, *Trichosporonaceae*, and *Aspergillaceae* (Figure 3d), were remarkably enriched in the rhizosphere soil. Moreover, both endophytic and rhizosphere soil bacteria of wild *G. pensilis* were significantly involved in terpenoid and polyketide metabolism. In addition, rhizosphere bacteria were enriched in processes such as membrane transport, translation, protein folding, sorting, and degradation (Supplementary Figures 1,c,d). It is noteworthy that saprotrophic fungi were notably abundant in the rhizosphere soil (Supplementary Figure 1f). Previous studies have claimed that *Aspergillus*, *Planctomycetota*, *Basidiomycota*, *Ascomycota*, *Tremellomycetes*, and microorganisms associated with terpenoid and polyketide metabolism can decompose plant and animal residues (Medina et al., 2020; Pfliegler et al., 2020; Chen et al., 2023; Tamang et al., 2023; Klimek et al., 2024; Manici et al., 2024), thereby enhancing soil nutrient availability, which may be a key factor contributing to the high nutrient content in wild *G. pensilis*. What’s more, *Hyphomicrobiaceae* is believed to enhance nitrogen fixation (Li et al., 2021), while saprophytic fungi such as *Oncopodiella*, *Eurotiales*, and *Aspergillaceae* accelerate carbon and nitrogen cycling (Wang and Bao, 2024; Du et al., 2024; Ma et al., 2024). The significant enrichment of these microorganisms may facilitate alleviate local nutrient limitations, enabling wild *G. pensilis* to efficiently acquire nutrients even without human intervention. Additionally, Song et al. (2023) pointed out that the enhancement of membrane transport pathways contributes to improving plant salt tolerance, while Xiao et al. (2021) suggested that the enrichment of folding, sorting, and degradation pathways is closely related to plant responses to environmental stress. We speculate that these metabolic pathways are crucial for the environmental adaptation of wild *G. pensilis* in harsh natural conditions.

Further observations reveal notable differences in the symbiotic networks of rhizosphere microbes across various habitats of *G. pensilis*. On one hand, cultivated *G. pensilis* roots and rhizosphere soil exhibit more complex bacterial networks, characterized by higher values in edge count, average degree, clustering coefficient, and network density when compared to wild ones (Figure 5a). Previous studies have emphasized the positive role of microbial network complexity in enhancing functional diversity (Chen et al., 2022). Interestingly, functional annotation results from this study show that the root and rhizosphere bacterial communities of cultivated *G. pensilis* are enriched with 5 and 11 metabolic pathways, respectively, which is significantly greater than the 1 and 6 pathways identified in wild ones (Supplementary Figures 1c,d). This suggests that the intricate bacterial networks in cultivated *G. pensilis* may contribute to its enhanced functional diversity. On the other hand, the fungal symbiotic networks in the roots and rhizosphere soil of wild *G. pensilis* display higher node count, edge count, average degree, and clustering coefficient, indicating a more complex overall structure (Figure 5b). Such complexity is known to accelerate litter decomposition and improve soil nutrient cycling efficiency (Raza et al., 2023; Cao et al., 2024; Ding et al., 2024). This may further elucidate the mechanism underlying the higher nutrient content observed in wild *G. pensilis*.

Land management practices can lead to soil disturbance, which diminishes the stability of microbial communities (Xu et al., 2019). Particularly, the high homogenization of plant habitats can render microbial communities occupying distinct ecological niches more sensitive and unstable in response to human cultivation and management activities (Schöps et al., 2018). Our findings further substantiated this observation. We discovered that biomarkers for rhizosphere bacteria, endophytic bacteria, and endophytic fungi were more abundant in cultivated *G. pensilis* compared to wild ones (Figure 3). To some extent, biomarkers serve as indicators of microbial sensitivity to external changes: the more biomarkers present, the higher the sensitivity (Chen et al., 2024). Agricultural practices, including tillage, fertilization, and management machinery, can disrupt the soil microbial environment (Mendes et al., 2015). Under environmental changes, the stability of soil microbial genomes may be threatened (Guo et al., 2018). Therefore, to enhance the stability of rhizosphere microbial communities in cultivated *G. pensilis*, it is essential to minimize habitat disturbances while promoting healthy plant growth. The study claimed that a higher P/N ratio can enhance nutrient use efficiency, but it may compromise ecological stability in extreme environments (Wang Y. et al., 2023). Furthermore, saprotrophic fungi are known to accumulate in decayed or diseased plants, thereby increasing the risk of plant diseases (Liu et al., 2021). In our research, both the P/N ratio in the rhizosphere microbial symbiotic network and the abundance of saprotrophic fungi were found to be greater in wild *G. pensilis* compared to cultivated ones. Besides, our field investigation revealed a relatively high incidence of disease among wild *G. pensilis*. These findings imply that an elevated P/N ratio and an increase in saprotrophic fungal abundance may indirectly influence the stability of *G. pensilis*.

Overall, cultivated *G. pensilis* enhances the degradation of organic matter and the release of nutrients by optimizing microbial functions. Simultaneously, it establishes a more complex bacterial symbiotic network, thereby improving resource utilization efficiency and ecological competitiveness. In contrast, wild *G. pensilis* relies on abundant saprotrophic fungi and a complex fungal symbiotic network to accelerate organic matter decomposition and nutrient acquisition, which enhances its environmental adaptability. Given the above information, our study emphasizes the importance of understanding plant-microbe interactions in the conservation of the endangered plant *G. pensilis*. Notably, researchers have utilized the plate streaking method to isolate and purify growth-promoting microorganisms for the conservation of endangered species such as *Pulsatilla tongkangensis*, *Glycyrrhiza glabra*, and *Pinus chiapensis*. Inoculating these microorganisms into seeds, seedling roots, or soil has significantly enhanced plant nutrient uptake efficiency, stress tolerance, and growth performance (Domínguez-Castillo et al., 2021; Dutta et al., 2021; Mousavi et al., 2022). Moreover, co-inoculation with symbiotic bacteria has optimized the rhizosphere symbiotic network, boosting microbial diversity and decrease disease risks (Lopes et al., 2021). Therefore, to more effectively *G. pensilis*, we can isolate and purify rhizosphere microorganisms with phosphorus-solubilizing, nitrogen-fixing, and IAA-producing capabilities, and co-inoculating them with symbiotic microbes into seedling roots to synergistically promote plant growth and health.

Plant growth is closely associated with rhizosphere microorganisms and the soil environment, and the physicochemical characteristics of the soil can influence the structure and function of rhizosphere microbial communities (Song et al., 2019; Islam et al., 2023). This relationship is also evident in the rhizosphere soil of *G. pensilis*. Our findings indicated that the rhizosphere microbial community of wild *G. pensilis* was closely associated with various soil environmental factors. The fungal microbial structure was significantly related to 15 soil environmental factors, while the bacterial microbial structure correlated with 7 factors (*p* < 0.05) (Supplementary Figures 2a,b). Notably, fungal functional abundance showed a strong correlation with TP, AP, and AK levels, whereas fungal alpha diversity was significantly correlated with LAP and SMC levels (*p* < 0.01). Similar results were observed in the rhizosphere of cultivated *G. pensilis*, where bacterial communities were significantly correlated with AP, pH, NN, BG, CBH, and NAG levels (*p* < 0.05). In contrast, fungal communities showed no significant correlation with soil-related indicators (*p* > 0.05) (Supplementary Figures 2a,b). These results suggested that, although the rhizosphere microbes of the cultivated *G. pensilis* were also closely related to soil environmental factors, similar to those of wild *G. pensilis*, the degree of association was lower, and fewer soil drivers were identified.

What’s more, when examining the correlation between all soil nutrients (including SOC, TN, TP, TK, AP, AK, AN, NN) and rhizosphere microorganisms, we discovered that phosphorus content was closely associated with the rhizosphere fungi of wild *G. pensilis*. Specifically, TP was significantly correlated with fungal function and diversity, while AP was significantly related to fungal function (*p* < 0.05). Previous studies have reported that the availability of phosphorus in the rhizosphere can influence plant-microbe symbiosis (Islam et al., 2023). In alkaline soils, where mineral phosphorus is not readily absorbed by plants, arbuscular mycorrhizal fungi can enhance phosphorus uptake in these plants (Begum et al., 2019; Etesami et al., 2021; Liu S. L. et al., 2024). This indicated that phosphorus cycling plays a specific role in the rhizosphere fungi of wild *G. pensilis*. These findings elucidate the mechanism by which wild *G. pensilis* depends on rhizosphere microbes to acquire nutrients and withstand external environmental factors.

Multivariate regression models and RF analysis further elucidated the effects of soil environmental factors on rhizosphere microbial communities (Figures 6a,b). Our results indicated that TP and TK levels significantly affected cultivated *G. pensilis*, with both factors being positively correlated with microbial diversity in the rhizosphere of cultivated *G. pensilis*. This suggested that the lower levels of TP and TK might have indirectly hindered the growth of cultivated *G. pensilis*. In contrast, the most important factor influencing the structure and function of rhizosphere microbes in wild *G. pensilis* was AK. In the soil environment of cultivated *G. pensilis*, macro-nutrients such as TK and TP are effectively supplemented due to human management interventions, which enhances the growth efficiency of cultivated *G. pensilis*. As one of the primary nutrients required for plant growth, potassium and phosphorus play a crucial role in the physiological metabolism and growth rate of plants (Wang et al., 2021). They influence the composition of root exudates, thereby providing specific carbon sources and energy for rhizosphere microorganisms. Consequently, the structure of the rhizosphere microbial community in cultivated *G. pensilis* is significantly regulated by the levels of TK and TP, and the supply of these nutrients promotes microbial diversity and activity, forming a rhizosphere microbial community more favorable for plant growth. In contrast, the wild *G. pensilis* grows in a natural soil environment with minimal human intervention, where nutrient levels are relatively limited. This leads them to develop symbiotic relationships with rhizosphere microorganisms to enhance nutrient acquisition efficiency. AK is the form of potassium that can be directly absorbed by plants and microorganisms, which is particularly significant for wild plants. In natural soils, wild plants rely on rhizosphere microorganisms to assist in obtaining essential nutrients like potassium (Soumare et al., 2023). Adequate potassium supply can significantly activate the metabolic activity of rhizosphere microorganisms; thus, variations in AK levels directly affect the function and diversity of rhizosphere microbes associated with wild *G. pensilis*. Overall, phosphorus and potassium were crucial for the growth of *G. pensilis*. Therefore, in practical cultivation, applying appropriate amounts of phosphorus and potassium fertilizers is vital for maintaining rhizosphere microbial diversity and enhancing plant adaptability.

Additionally, it has been reported that some pathogens are introduced into the soil through feces, water, and waste, subsequently colonizing plant roots and inhabiting various plant organs via the vascular system (Sobiczewski and Iakimova, 2022). Human pathogens have been identified in plant leaves and rhizospheres, and high levels of human disease-associated metabolic activities may increase the likelihood of plant diseases (Huang et al., 2019). In this study, metabolic pathways related to human diseases were negatively correlated with soil environmental factors in the cultivated *G. pensilis*, except for NN. This indicated that more fertile soil for cultivated *G. pensilis* is associated with a lower likelihood of disease. Conversely, an increase in NN in the soil could promote disease in the cultivated *G. pensilis*. In wild *G. pensilis*, bacteria related to human disease metabolism were positively correlated with potassium content in the rhizosphere soil (including TK and AK) but negatively correlated with soil enzyme activity, SOC, and SMC levels. Therefore, to mitigate the risk of plant diseases, it is crucial to minimize soil contamination within the *G. pensilis* ecosystem. Besides, the management of human-controlled soil nutrient availability should be prioritized, which included reducing nitrogen fertilizer application for cultivated *G. pensilis* and increasing SMC for wild *G. pensilis*, among other measures.

## 5 Conclusion

In conclusion, significant differences were observed in the composition, diversity, and function of rhizospheric soil microbial communities between wild and cultivated *G. pensilis* plants. Mantel analysis revealed a significant association between the fungal microbial structure and 15 soil environmental factors, while the bacterial microbial structure was highly correlated with 7 soil environmental factors. Notably, the rhizospheric bacterial communities of cultivated *G. pensilis* were significantly correlated only with AP, pH, NN, BG, CBH, NAG and SMC. RF analysis identified TP and TK as the vital influencing factors for rhizospheric microbes in cultivated *G. pensilis*, while AK was crucial for the wild *G. pensilis* microbes. Overall, these findings highlight the differences in rhizospheric microbes between wild and cultivated *G. pensilis*, which may be attributed to the differences in land use management practices and soil environmental factors. Moreover, the results of this study will provide valuable references for the sustainable growth and restoration of the rare and endangered plant *G. pensilis*.

## Data Availability

The original contributions presented in the study are publicly available. This data can be found here: NCBI BioProject PRJNA1235944.

## References

[ref1] Bao S. D. (2000). Soil agrochemical analysis. Beijing: China Agricultural Press.

[ref2] Begum N. Qin C. Ahanger M. A. Raza S. Khan M. I. Ashraf M.. (2019). Role of arbuscular mycorrhizal fungi in plant growth regulation: implications in abiotic stress tolerance. Front. Plant Sci. 10:1068. doi: 10.3389/fpls.2019.01068, PMID: 31608075 PMC6761482

[ref3] Cao T. T. Zhang Q. Chen Y. R. Li Q. Fang Y. Luo Y. C.. (2024). Enlarging interface reverses the dominance of fungi over bacteria in litter decomposition. Soil Biol. Biochem. 198:109543. doi: 10.1016/j.soilbio.2024.109543

[ref4] Chen Q. Y. Liu Z. J. Zhou J. B. Xu X. P. Zhu Y. J. (2021). Long-term straw mulching with nitrogen fertilization increases nutrient and microbial determinants of soil quality in a maize–wheat rotation on China’s loess plateau. Sci. Total Environ. 775:145930. doi: 10.1016/j.scitotenv.2021.145930

[ref5] Chen W. Q. Wang J. Y. Chen X. Meng Z. X. Xu R. Duoji D. Z.. (2022). Soil microbial network complexity predicts ecosystem function along elevation gradients on the Tibetan plateau. Soil Boil. Biochem. 172:108766. doi: 10.1016/j.soilbio.2022.108766

[ref6] Chen D. Wang C. Ma X. F. Chen K. P. Wang Z. T. Wang Q.. (2023). Dynamic changes in soil fungal communities and functional groups in response to sugarcane/soybean intercropping with reduced nitrogen fertilizer application. Biol. Fertil. 59, 363–378. doi: 10.1007/s00374-023-01709-5

[ref7] Chen J. Xu D. L. Xiao Q. C. Zheng Y. X. Liu H. J. Li X. Y.. (2024). Responses of soil microbial diversity, network complexity and multifunctionality to environments changes in volcanic ecosystems. J. Environ. 12:113334. doi: 10.1016/j.jece.2024.113334

[ref8] Deng Y. Kong W. Y. Zhang X. M. Zhu Y. Xie T. Chen M.. (2024). Rhizosphere microbial community enrichment processes in healthy and diseased plants: implications of soil properties on biomarkers. Front. Microbiol. 15:1333076. doi: 10.3389/fmicb.2024.1333076, PMID: 38505554 PMC10949921

[ref9] Dhungana I. Kantar M. B. Nguyen N. H. (2023). Root exudate composition from different plant species influences the growth of rhizosphere bacteria. Rhizosphere 25:100645:100645. doi: 10.1016/j.rhisph.2022.100645

[ref10] Ding Y. H. Gao X. D. Shu D. T. Siddique K. H. Song X. L. Wu P. T.. (2024). Enhancing soil health and nutrient cycling through soil amendments: improving the synergy of bacteria and fungi. Sci. Total Environ. 923:171332. doi: 10.1016/j.scitotenv.2024.171332, PMID: 38447716

[ref11] Domínguez-Castillo C. Alatorre-Cruz J. M. Castañeda-Antonio D. Munive J. A. Guo X. López-Olguín J. F.. (2021). Potential seed germination-enhancing plant growth-promoting rhizobacteria for restoration of *Pinus chiapensis* ecosystems. J. For. Res. 32, 2143–2153. doi: 10.1007/s11676-020-01250-3

[ref12] Du J. H. Yu Y. F. Tang C. C. Zong K. K. Zhang S. J. Zhang Q. Q.. (2024). Organic fertilizers increase the proportion of saprotrophs favoring soil nitrification under medicinal plants *Fritillaria thunbergii*. Ind. Crop. Prod. 219:119129. doi: 10.1016/j.indcrop.2024.119129

[ref13] Dutta S. Na C. S. Lee Y. H. (2021). Features of bacterial microbiota in the wild habitat of *Pulsatilla tongkangensis*, the endangered "long-sepal Donggang Pasque-Flower Plant," endemic to karst topography of Korea. Front. Microbiol. 12:656105. doi: 10.3389/fmicb.2021.656105, PMID: 34305828 PMC8297415

[ref14] Etesami H. Jeong B. R. Glick B. R. (2021). Contribution of arbuscular mycorrhizal fungi, phosphate–solubilizing bacteria, and silicon to P uptake by plant. Front. Plant Sci. 12:69961. doi: 10.3389/fpls.2021.699618, PMID: 34276750 PMC8280758

[ref17] Gao J. X. Zhang F. B. (2023). Influence of companion planting on microbial compositions and their symbiotic network in pepper continuous cropping soil. J. Microbiol. Biotechnol. 33, 760–770. doi: 10.4014/jmb.2211.11032, PMID: 37072683 PMC10331949

[ref18] Gonçalves O. S. Fernandes A. S. Tupy S. M. Ferreira T. G. Almeida L. N. Creevey C. J.. (2024). Insights into plant interactions and the biogeochemical role of the globally widespread acidobacteriota phylum. Soil Biol. Biochem. 192:109369. doi: 10.1016/j.soilbio.2024.109369

[ref19] Guo Y. Q. Chen X. T. Wu Y. Y. Zhang L. Cheng J. M. Wei G. H.. (2018). Natural revegetation of a semiarid habitat alters taxonomic and functional diversity of soil microbial communities. Sci. Total Environ. 635, 598–606. doi: 10.1016/j.scitotenv.2018.04.171, PMID: 29679832

[ref20] Guo Y. X. Ren C. J. Yi J. J. Doughty R. Zhao F. Z. (2020). Contrasting responses of rhizosphere bacteria, fungi and arbuscular mycorrhizal fungi along an elevational gradient in a temperate montane forest of China. Front. Microbiol. 11:2042. doi: 10.3389/fmicb.2020.02042, PMID: 32973736 PMC7469537

[ref21] He D. X. Singh S. K. Peng L. Kaushal R. Vílchez J. I. Shao C. Y.. (2022). Flavonoid-attracted *Aeromonas* sp. from the Arabidopsis root microbiome enhances plant dehydration resistance. ISME J. 16, 2622–2632. doi: 10.1038/s41396-022-01288-7, PMID: 35842464 PMC9561528

[ref22] Huang X. Q. Zhou X. Zhang J. B. Cai Z. C. (2019). Highly connected taxa located in the microbial network are prevalent in the rhizosphere soil of healthy plant. Biol. Fert. Soils 55, 299–312. doi: 10.1007/s00374-019-01350-1

[ref23] Husna Tuheteru F. D. Arif A. (2021). The potential of arbuscular mycorrhizal fungi to conserve *Kalappia celebica*, an endangered endemic legume on gold mine tailings in Sulawesi, Indonesia. J. For. Res. 32, 675–682. doi: 10.1007/s11676-020-01097-8, PMID: 40052060

[ref24] Huy Thai T. Paoli M. Thi Hien N. Quang Hung N. Bighelli A. Casanova J.. (2023). Combined analysis by GC (RI), GC-MS and 13C NMR of leaf and wood essential oils from vietnamese *Glyptostrobus pensilis* (Staunton ex D. Don) K. Koch. Compounds 3, 447–458. doi: 10.3390/compounds3030033

[ref25] Islam S. U. Mangral Z. A. Hussain K. Tariq L. Bhat B. A. Khuroo A. A.. (2023). Unravelling diversity, drivers, and indicators of soil microbiome of *Trillium govanianum*, an endangered plant species of the Himalaya. Environ. Res. 227:115819. doi: 10.1016/j.envres.2023.115819, PMID: 37011799

[ref26] Kalia V. C. Gong C. Patel S. K. Lee J. K. (2021). Regulation of plant mineral nutrition by signal molecules. Microorganisms 9:774. doi: 10.3390/microorganisms9040774, PMID: 33917219 PMC8068062

[ref27] Klimek D. Herold M. Calusinska M. (2024). Comparative genomic analysis of Planctomycetota potential for polysaccharide degradation identifies biotechnologically relevant microbes. BMC Genomics 25:523. doi: 10.1186/s12864-024-10413-z, PMID: 38802741 PMC11131199

[ref28] Lei J. Wu H. B. Li X. Y. Guo W. F. Duan A. G. Zhang J. G. (2023). Response of rhizosphere bacterial communities to near-natural forest management and tree species within Chinese fir plantations. Microbiol. Spectr. 11:e02328-22. doi: 10.1128/spectrum.02328-22, PMID: 36688690 PMC9927156

[ref29] Li G. T. Gong P. F. Zhou J. Wang L. Song X. Ding P. H.. (2024). The succession of rhizosphere microbial community in the continuous cropping soil of tobacco. Front. Environ. Sci. 11:1251938. doi: 10.3389/fenvs.2023.1251938

[ref30] Li F. R. Lu S. G. Sun W. B. (2024). Comparison of rhizosphere bacterial communities of *Pinus squamata*, a plant species with extremely small populations (PSESP) in different conservation sites. Microorganisms 12:638. doi: 10.3390/microorganisms12040638, PMID: 38674583 PMC11051972

[ref32] Li J. Y. Tang G. D. Liu H. W. Luo X. Y. Wang J. T. (2025). Characterizing the microbiome recruited by the endangered plant *Firmiana danxiaensis* in phosphorus-deficient acidic soil. Front. Microbiol. 15:1439446. doi: 10.3389/fmicb.2024.1439446, PMID: 39881984 PMC11774962

[ref33] Li Y. L. Wang X. Y. Chen X. Y. Lu J. Y. Jin Z. X. Li J. M. (2023). Functions of arbuscular mycorrhizal fungi in regulating endangered species *Heptacodium miconioides* growth and drought stress tolerance. Plant Cell Rep. 42, 1967–1986. doi: 10.1007/s00299-023-03076-9, PMID: 37812279

[ref34] Li Y. L. Wang Y. M. Shen C. Xu L. Yi S. Q. Zhao Y. L.. (2021). Structural and predicted functional diversities of bacterial microbiome in response to sewage sludge amendment in coastal mudflat soil. Biology 10:1302. doi: 10.3390/biology10121302, PMID: 34943217 PMC8698727

[ref35] Liu S. L. Li H. M. Xie X. Y. Chen Y. X. Lang M. Chen X. (2024). Long-term moderate fertilization increases the complexity of soil microbial community and promotes regulation of phosphorus cycling genes to improve the availability of phosphorus in acid soil. Appl. Soil Ecol. 194:105178:105178. doi: 10.1016/j.apsoil.2023.105178

[ref36] Liu Q. W. Wang S. X. Li K. Qiao J. Guo Y. S. Liu Z. D.. (2021). Responses of soil bacterial and fungal communities to the long-term monoculture of grapevine. Appl. Microbiol. Biot. 105, 7035–7050. doi: 10.1007/s00253-021-11542-1, PMID: 34477939

[ref37] Liu D. L. Xu L. Q. Wang H. Wang X. Song B. Q. Wang Q. H. (2024). Root exudates promoted microbial diversity in the sugar beet rhizosphere for organic nitrogen mineralization. Agriculture 14:1094. doi: 10.3390/agriculture14071094

[ref38] Liu J. X. Zeng D. J. Huang Y. Zhong L. S. Liao J. L. Shi Y. X.. (2024). The structure and diversity of bacteria and fungi in the roots and rhizosphere soil of three different species of *Geodorum*. BMC Genomics 25:222. doi: 10.1186/s12864-024-10143-2, PMID: 38418975 PMC10903027

[ref39] Lopes M. J. D. S. Dias-Filho M. B. Gurgel E. S. C. (2021). Successful plant growth-promoting microbes: inoculation methods and abiotic factors. Front. Sustainable Food Syst. 5:606454. doi: 10.3389/fsufs.2021.606454, PMID: 40046924

[ref40] Ma Y. Y. Shen Y. Zhou X. P. Ma H. B. Lan J. Fu B. Z.. (2024). Biological decline of alfalfa is accompanied by negative succession of rhizosphere soil microbial communities. Plan. Theory 13:2589. doi: 10.3390/plants13182589, PMID: 39339564 PMC11434760

[ref41] Manici L. M. Caputo F. De Sabata D. Fornasier F. (2024). The enzyme patterns of Ascomycota and Basidiomycota fungi reveal their different functions in soil. Appl. Soil Ecol. 196:105323:105323. doi: 10.1016/j.apsoil.2024.105323

[ref42] Medina J. Monreal C. M. Orellana L. Calabi-Floody M. González M. E. Meier S.. (2020). Influence of saprophytic fungi and inorganic additives on enzyme activities and chemical properties of the biodegradation process of wheat straw for the production of organo-mineral amendments. J. Environ. Manag. 255:109922. doi: 10.1016/j.jenvman.2019.109922, PMID: 32063309

[ref43] Mendes L. W. Tsai S. M. Navarrete A. A. Hollander M. D. Veen J. A. Kuramae E. E. (2015). Soil-borne microbiome: linking diversity to function. Microb. Ecol. 70, 255–265. doi: 10.1007/s00248-014-0559-2, PMID: 25586384

[ref44] Mofini M. T. Diedhiou A. G. Simonin M. Dondjou D. T. Pignoly S. Ndiaye C.. (2022). Cultivated and wild pearl millet display contrasting patterns of abundance and co-occurrence in their root mycobiome. Sci. Rep. 12:207. doi: 10.1038/s41598-021-04097-8, PMID: 34997057 PMC8741948

[ref45] Mousavi S. S. Karami A. Saharkhiz M. J. Etemadi M. Ravanbakhsh M. (2022). Microbial amelioration of salinity stress in endangered accessions of Iranian licorice (*Glycyrrhiza glabra* L.). BMC Plant Biol. 22:322. doi: 10.1186/s12870-022-03703-9, PMID: 35790900 PMC9254424

[ref46] Nakayasu M. Takamatsu K. Yazaki K. Sugiyama A. (2023). Plant specialized metabolites in the rhizosphere of tomatoes: secretion and effects on microorganisms. Biosci. Biotechno. Bioche. 87, 13–20. doi: 10.1093/bbb/zbac181, PMID: 36373409

[ref47] Narsing Rao M. P. Lohmaneeratana K. Bunyoo C. Thamchaipenet A. (2022). Actinobacteria–plant interactions in alleviating abiotic stress. Plan. Theory 11:2976. doi: 10.3390/plants11212976, PMID: 36365429 PMC9658302

[ref48] Ni X. Y. Zhao C. Y. Li J. S. Li B. Zhu J. F. (2023). Comparison of bacterial diversity in the rhizosphere of *Chromolaena odorata* (L.) RM king and H. Rob. in different habitats. Sustainability 15:2315. doi: 10.3390/su15097104, PMID: 39857420

[ref49] Parasar B. J. Sharma I. Agarwala N. (2024). Root exudation drives abiotic stress tolerance in plants by recruiting beneficial microbes. App. Soil Ecol. 198:105351:105351. doi: 10.1016/j.apsoil.2024.105351

[ref50] Park I. Seo Y. S. Mannaa M. (2023). Recruitment of the rhizo-microbiome army: assembly determinants and engineering of the rhizosphere microbiome as a key to unlocking plant potential. Front. Microbiol. 14:1163832. doi: 10.3389/fmicb.2023.1163832, PMID: 37213524 PMC10196466

[ref51] Pfliegler W. P. Pócsi I. Győri Z. Pusztahelyi T. (2020). The *Aspergilli* and their mycotoxins: metabolic interactions with plants and the soil biota. Front. Microbiol. 10:2921. doi: 10.3389/fmicb.2019.02921, PMID: 32117074 PMC7029702

[ref52] Phong N. V. Trang N. M. Quyen C. T. Anh H. L. T. Vinh L. B. (2022). SARS-CoV-2 main protease and papain-like protease inhibition by abietane-type diterpenes isolated from the branches of *Glyptostrobus pensilis* using molecular docking studies. Nat. Prod. Res. 36, 6336–6343. doi: 10.1080/14786419.2022.2025801, PMID: 35021907

[ref53] Pueyo-Herrera P. Tang C. Q. Matsui T. Ohashi H. Qian S. H. Yang Y. C.. (2023). Ecological niche modeling applied to the conservation of the east Asian relict endemism *Glyptostrobus pensilis* (Cupressaceae). New For. 54, 1131–1152. doi: 10.1007/s11056-022-09960-8

[ref54] Rakitin A. L. Kulichevskaya I. S. Beletsky A. V. Mardanov A. V. Dedysh S. N. Ravin N. V. (2024). Verrucomicrobia of the family Chthoniobacteraceae participate in xylan degradation in boreal peat soils. Microorganisms 12:2271. doi: 10.3390/microorganisms12112271, PMID: 39597660 PMC11596606

[ref55] Raza T. Qadir M. F. Khan K. S. Eash N. S. Yousuf M. Chatterjee S.. (2023). Unrevealing the potential of microbes in decomposition of organic matter and release of carbon in the ecosystem. J. Environ. Manag. 344:118529. doi: 10.1016/j.jenvman.2023.118529, PMID: 37418912

[ref56] Schöps R. Goldmann K. Herz K. Lentendu G. Schöning I. Bruelheide H.. (2018). Land-use intensity rather than plant functional identity shapes bacterial and fungal rhizosphere communities. Front. Microbiol. 9:2711. doi: 10.3389/fmicb.2018.02711, PMID: 30515138 PMC6255942

[ref57] Sobiczewski P. Iakimova E. T. (2022). Plant and human pathogenic bacteria exchanging their primary host environments. J. Hortic. Res. 30, 11–30. doi: 10.2478/johr-2022-0009

[ref58] Song J. Han Y. Y. Bai B. X. Jin S. He Q. F. Ren J. H. (2019). Diversity of arbuscular mycorrhizal fungi in rhizosphere soils of the Chinese medicinal herb *Sophora flavescens* Ait. Soil. Till. Res. 195:104423. doi: 10.1016/j.still.2019.104423

[ref59] Song P. P. Liu J. L. Huang P. Han Z. L. Wang D. L. Sun N. X. (2023). Diversity and structural analysis of rhizosphere soil microbial communities in wild and cultivated *Rhizoma Atractylodis Macrocephalae* and their effects on the accumulation of active components. PeerJ 11:e14841. doi: 10.7717/peerj.14841, PMID: 36811005 PMC9939024

[ref60] Soumare A. Djibril S. A. R. R. Diédhiou A. G. (2023). Potassium sources, microorganisms and plant nutrition: challenges and future research directions. Pedosphere 33, 105–115. doi: 10.1016/j.pedsph.2022.06.025

[ref61] Tamang A. Swarnkar M. Kumar P. Kumar D. Pandey S. S. Hallan V. (2023). Endomicrobiome of in vitro and natural plants deciphering the endophytes-associated secondary metabolite biosynthesis in *Picrorhiza kurrooa*, a Himalayan medicinal herb. Microbiol. Spectr. 11:e02279-23. doi: 10.1128/spectrum.02279-23, PMID: 37811959 PMC10715050

[ref62] Tang C. Q. Yang Y. C. Momohara A. Wang H. C. Luu H. T. Li S. F.. (2019). Forest characteristics and population structure of *Glyptostrobus pensilis*, a globally endangered relict species of southeastern China. Plant Divers 41, 237–249. doi: 10.1016/j.pld.2019.06.007, PMID: 31528783 PMC6742968

[ref64] Tong X. X. Wang K. Y. Chen Z. H. Wang L. L. Xiang T. H. (2021). Endangerment of *Ostrya rehderiana* Chun and its relationship with rhizosphere soil microflora. Agron. J. 113, 746–759. doi: 10.1002/agj2.20451

[ref65] Wang Q. C. Bao H. Y. (2024). Research on the mechanism of root endophytes of *Morus alba* L. and *Fraxinus mandshurica* Rupr., two host plants growing *Inonotus hispidus* (bull.) P. Karst., with metabarcoding and metabolomics. Horticulturae 10:1074. doi: 10.3390/horticulturae10101074, PMID: 39857420

[ref66] Wang B. T. Chen H. M. Qu P. Lin R. He S. M. Li W. F.. (2023). Effect of different cultivation patterns on *Amomum villosum* yield and quality parameters, rhizosphere soil properties, and rhizosphere soil microbes. Horticulturae 9:306. doi: 10.3390/horticulturae9030306

[ref67] Wang Y. Chen Y. F. Wu W. H. (2021). Potassium and phosphorus transport and signaling in plants. J. Integr. Plant Biol. 63, 34–52. doi: 10.1111/jipb.13053, PMID: 33325114

[ref68] Wang Y. Ma Q. Y. Wang L. L. Hu J. K. Xue H. Y. Han D. F.. (2023). Structure and function analysis of cultivated *Meconopsis integrifolia* soil microbial community based on high-throughput sequencing and culturability. Biology 12:160. doi: 10.3390/biology12020160, PMID: 36829439 PMC9952792

[ref69] Wu X. T. Ruhsam M. Wen Y. F. Thomas P. I. Worth J. R. P. Lin X. Y.. (2020). The last primary forests of the tertiary relict *Glyptostrobus pensilis* contain the highest genetic diversity. Forestry 93, 359–375. doi: 10.1093/forestry/cpz063

[ref70] Xiao Y. Q. Jiang R. H. Wu X. X. Zhong Q. Li Y. Wang H. Q. (2021). Comparative genomic analysis of *Stenotrophomonas maltophilia* strain W18 reveals its adaptative genomic features for degrading polycyclic aromatic hydrocarbons. Microbiol. Spectr. 9:e01420-21. doi: 10.1128/Spectrum.01420-21, PMID: 34817285 PMC8612148

[ref71] Xie B. Chen Y. H. Cheng C. G. Ma R. P. Zhao D. Y. Li Z.. (2022). Long-term soil management practices influence the rhizosphere microbial community structure and bacterial function of hilly apple orchard soil. Appl. Soil Ecol. 180:104627. doi: 10.1016/j.apsoil.2022.104627

[ref72] Xiong J. Hu C. L. Wang P. P. Gao D. D. Huang F. Li J.. (2020). Spirobiflavonoid stereoisomers from the endangered conifer *Glyptostrobus pensilis* and their protein tyrosine phosphatase 1B inhibitory activity. Bioorg. Med. Chem. Lett. 30:126943. doi: 10.1016/j.bmcl.2019.126943, PMID: 31924496

[ref73] Xu S. Q. Tian L. Chang C. L. Li X. J. Tian C. J. (2019). Cultivated rice rhizomicrobiome is more sensitive to environmental shifts than that of wild rice in natural environments. Appl. Soil Ecol. 140, 68–77. doi: 10.1016/j.apsoil.2019.04.006

[ref74] Yao Z. T. Khan A. Xu Y. Z. Pan K. Y. Zhang M. Q. (2024). Profiling of rhizosphere bacterial community associated with sugarcane and banana rotation system. Chem. Biol. Technol. Agric. 11:91. doi: 10.1186/s40538-024-00616-7

[ref76] Ye E. Z. Zhang M. Z. Yang Q. Y. Ye L. Q. Liu Y. P. Zhang G. F.. (2022). Prediction of suitable distribution of a critically endangered plant *Glyptostrobus pensilis*. Forests 13:257. doi: 10.3390/f13020257

[ref77] You Y. M. Wang L. R. Liu X. T. Wang X. L. Jiang L. P. Ding C. J.. (2024). Interspecific plant interaction structures the microbiomes of poplar-soil interface to alter nutrient cycling and utilization. Microbiol. Spectr. 12:e0336823. doi: 10.1128/spectrum.03368-23, PMID: 38197657 PMC10846221

[ref78] Yuan L. Ma S. Y. Liu K. Wang T. Chen L. (2023). High frequency adventitious shoot regeneration from hypocotyl-derived callus of *Glyptostrobus pensilis*, a critically endangered plant. Plant Cell Tiss. Org. 152, 139–149. doi: 10.1007/s11240-022-02396-0

[ref80] Zhang Y. Z. Zheng Y. W. Gong Q. H. Fu S. Q. Chen C. Tang Y. J.. (2024). Human impacts on Holocene vegetation and wetland degradation in the lower Pearl River, Southern China. Land 13:530. doi: 10.3390/land13040530, PMID: 39857420

[ref81] Zhang K. Zheng D. F. Gu Y. Xu J. Wang M. Y. Mu B.. (2024). Utilizing soil organic phosphorus for sustainable crop production: insights into the rhizosphere. Plant Soil 498, 57–75. doi: 10.1007/s11104-023-06136-x

[ref82] Zhang C. Zhou L. T. Wang Y. Y. Lin R. Y. Wu Z. Y. (2024). Functional characteristics of rhizosphere soil microbial communities of relict plant *Glyptostrobus pensilis* in different seasons. For. Res. 37, 1–11. doi:doi: 10.12403/j.1001-1498.20230221

[ref83] Zhou N. Mei C. M. M. Zhu X. Y. Zhao J. J. Ma M. G. Li W. D. (2022). Research progress of rhizosphere microorganisms in *Fritillaria L.* medicinal plants. Front. Bioeng. Biotechnol. 10:1054757. doi: 10.3389/fbioe.2022.1054757, PMID: 36420438 PMC9676442

[ref84] Zhou X. G. Zhu H. G. Wen Y. G. Goodale U. M. Zhu Y. L. Yu S. F.. (2020). Intensive management and declines in soil nutrients lead to serious exotic plant invasion in *Eucalyptus* plantations under successive short-rotation regimes. Land Degrad. Dev. 31, 297–310. doi: 10.1002/ldr.3449

[ref85] Zhu X. M. Kaal J. Traoré M. Kuang Y. W. (2024). Characterization of modern and waterlogged archaeological cypress (*Glyptostrobus pensilis*) wood: an analytical pyrolysis (Py-GC-MS and THM-GC-MS) and infrared spectroscopy (FTIR-ATR) study of within tree (radial) and decay-induced compositional variations. J. Anal. Appl. Pyrolysis 177:106347:106347. doi: 10.1016/j.jaap.2023.106347

[ref86] Zuo Y. W. He P. Zhang J. H. Li W. Q. Ning D. H. Zeng Y. L.. (2022a). Contrasting responses of multispatial soil fungal communities of *Thuja sutchuenensis* Franch., an extremely endangered conifer in Southwestern China. Microbiol. Spectr. 10:e00260-22. doi: 10.1128/spectrum.00260-22, PMID: 35735985 PMC9431436

[ref87] Zuo Y. W. Yu F. Q. Zhang J. H. Xia C. Y. Zhang H. Deng H. P. (2022b). Contrasting responses of rhizosphere fungi of *Scutellaria tsinyunensis*, an endangered plant in southwestern China. Microbiol. Spectr. 10:e00225-22. doi: 10.1128/spectrum.00225-22, PMID: 35863021 PMC9430849

