# Supplementary material for: Differences in rhizospheric microbial communities between cultivated and wild endangered Glyptostrobus pensilis
Source: Front Microbiol. 2025 Mar 27;16:1548836. doi: 10.3389/fmicb.2025.1548836 (PMC11983652; doi:10.3389/fmicb.2025.1548836)
Supplement: Supplementary file 1 [file Data_Sheet_1.docx]

Supplementary Material

# Supplementary Tables

**Supplementary Table 1.** Geographic locations, *Glyptostrobus pensilis* types, and sample numbers.

| Location | Longitude | Latitude | *G. pensilis* Type |
| --- | --- | --- | --- |
| Tiandeng County, Chongzuo City | 106°58'30″ E | 22°55'41″ N | Cultivated *G. pensilis* |
| Tiandeng County, Chongzuo City | 106°58'45″ E | 22°55'10″ N | Cultivated *G. pensilis* |
| Pingle County, Guilin City | 110°42'36″ E | 24°51'36″ N | Cultivated *G. pensilis* |
| Tiandeng County, Chongzuo City | 106°54'0″ E | 22°54'0″ N | Wild *G. pensilis* |
| Yanshan District, Guilin City | 110°19'48″ E | 25°2'24″ N | Wild *G. pensilis* |
| Cangwu County, Wuzhou City | 110°58'48″ E | 23°29'24″ N | Wild *G. pensilis* |
| Binyang County, Nanning City | 108°42'0″ E | 23°12'0″ N | Wild *G. pensilis* |
| Pingle County, Guilin City | 110°42'36″ E | 24°51'36″ N | Wild *G. pensilis* |
| Qintang District, Guigang City | 109°24'0″ E | 23°6'0″ N | Wild *G. pensilis* |

**Supplementary Table 2.** The environmental factors of rhizosphere microorganisms.

| Sample | SMC % | pH | SOC g/kg | TN g/kg | TP g/kg | TK g/kg | AN mg/kg | NN mg/kg |
| --- | --- | --- | --- | --- | --- | --- | --- | --- |
| CP | 14.63±1.65f | 4.91±0.19e | 8.68±0.24f | 0.78±0.05g | 0.49±0.04e | 12.31±0.28f | 10.44±6.90d | 4.79±1.05d |
| CT1 | 48.11±1.19c | 8.00±0.06ab | 7.77±0.16f | 0.82±0.04g | 0.58±0.02d | 14.87±0.17d | 8.24±0.56de | 0.19±0.12e |
| CT2 | 102.99±12.08a | 7.53±0.05c | 40.38±0.68b | 3.97±0.13b | 1.19±0.02b | 23.59±0.37b | 264.05±2.16a | 0.22±0.09e |
| WP | 20.77±1.32ef | 7.77±0.01bc | 20.42±0.84e | 1.79±0.06e | 1.09±0.01c | 17.36±0.25c | 1.04±0.14e | 51.87±0.28a |
| WT | 96.84±8.09a | 7.82±0.03b | 62.21±2.56a | 6.37±0.10a | 1.16±0.04b | 12.17±0.15f | 181.82±1.78b | 0.34±0.03e |
| WY | 27.96±2.08e | 7.86±0.05b | 36.20±0.27c | 3.30±0.07c | 1.61±0.02a | 9.27±0.17g | 8.41±0.36de | 0.35±0.19e |
| WB | 38.03±2.71d | 6.78±0.02d | 30.44±1.75d | 2.24±0.09d | 1.15±0.03bc | 31.16±0.52a | 0.77±0.19e | 32.32±2.70c |
| WC | 74.36±9.76b | 5.08±0.05e | 19.13±0.61e | 1.56±0.07f | 0.53±0.01de | 23.99±0.52b | 5.02±0.47de | 42.99±0.58b |
| WQ | 23.46±0.34ef | 8.24±0.07a | 8.50±0.20f | 0.82±0.02g | 0.39±0.02f | 14.10±0.31e | 49.43±2.12c | 0.77±0.11e |
|  |  |  |  |  |  |  |  |  |
| Sample | AP mg/kg | AK mg/kg | BG nmol/g/h | CBH nmol/g/h | NAG nmol/g/h | LAP nmol/g/h | ALP nmol/g/h |  |
| CP | 13.08±7.22d | 41.5±4.32g | 7.86±2.7g | 1.95±1.09gh | 8.05±1.65g | 20.46±4.84g | 35.53±3.61de |  |
| CT1 | 7.90±0.17def | 32.33±0.58h | 31.81±1.32c | 16.14±0.59c | 18.06±0.68e | 270.82±2.59a | 51.11±1.43c |  |
| CT2 | 66.03±2.00b | 111.67±11.06de | 43.35±2.02a | 19.42±0.76b | 21.30±1.60d | 106.22±3.63e | 34.95±2.57ef |  |
| WP | 65.03±0.51b | 213.33±4.04b | 27.01±0.34d | 11.13±0.16e | 17.07±0.68e | 175.48±8.03c | 29.72±0.30f |  |
| WT | 12.47±0.15de | 118.67±1.15d | 46.65±1.04a | 21.52±0.97a | 28.31±0.98c | 256.64±5.61b | 80.48±2.58a |  |
| WY | 31.90±0.36c | 105.67±1.53e | 3.30±0.15h | 0.77±0.06h | 5.10±0.03h | 64.70±3.03f | 13.28±0.12g |  |
| WB | 88.29±1.09a | 496.00±1.73a | 13.97±0.66f | 2.80±0.09g | 10.74±0.35f | 270.23±11.19a | 30.18±0.86ef |  |
| WC | 2.67±0.07f | 85.33±1.53f | 18.92±1.11e | 6.78±0.56f | 61.73±1.47a | 121.47±5.39d | 60.02±2.80b |  |
| WQ | 3.67±0.12ef | 150.00±0.00c | 35.91±2.09b | 13.72±0.59d | 31.79±1.43b | 23.69±1.31g | 40.87±2.57d |  |

Note: SMC, soil moisture content; SOC, soil organic carbon; TN, total nitrogen; TP, total phosphorus; TK, total potassium; AN, ammonium nitrogen; NN, nitrate nitrogen; AP, available phosphorus; AK, available potassium; BG, β-1,4-glucosidase; CBH, β-D-glucosidase; NAG, β-1,4-N-acetylglucosaminidase; LAP, leucine aminopeptidase; ALP, alkaline phosphatase. CP: Cultivated *G. pensilis* samples collected in Pingle County; CT1, CT2: Cultivated *G. pensilis* samples collected in Tiandeng County; WP, WT, WY, WB, WC, WQ: Wild *G. pensilis* samples collected in Pingle County, Tiandeng County, Yanshan District, Binyang County, Cangwu County, and Qintang District. Different letters in the same column indicate signifcant diference at the 0.05 level.

**Supplementary Table 3.** The diversity indices of bacterial and fungal communities.

| Sample | Bacteria | | | | | |
| --- | --- | --- | --- | --- | --- | --- |
|  | Root | | | Soil | | |
|  | Chao1 | Shannon | Simpson | Chao1 | Shannon | Simpson |
| CP | 2015.88±972.27a | 8.03±2.15ab | 0.92±0.17a | 1718.89±297.59cd | 7.98±1.05bc | 0.96±0.03ab |
| CT1 | 1253.86±409.72a | 6.11±1.42ab | 0.90±0.06a | 2143.79±154.35c | 8.97±0.55abc | 0.98±0.01ab |
| CT2 | 2293.12±413.82a | 9.05±1.42ab | 0.99±0.02a | 2876.09±72.32a | 10.07±0.18a | 0.99±0.00a |
| WP | 1874.98±575.12a | 8.20±0.57ab | 0.98±0.01a | 2796.25±833.57ab | 9.62±0.62a | 0.99±0.01a |
| WT | 2323.18±99.06a | 9.82±0.20a | 1.00±0.00a | 2938.65±309.07a | 9.95±0.12a | 0.99±0.00a |
| WY | 1717.43±145.24a | 8.43±0.70ab | 0.98±0.01a | 2247.77±26.9bc | 8.55±0.81abc | 0.97±0.02ab |
| WB | 2509.42±649.82a | 9.57±0.17a | 1.00±0.00a | 1853.72±105.83cd | 9.28±0.15ab | 0.99±0.00a |
| WC | 1429.41±262.70a | 7.26±0.42ab | 0.96±0.03a | 3022.53±71.75a | 9.21±0.87ab | 0.97±0.02ab |
| WQ | 1583.89±187.57a | 8.01±0.65ab | 0.97±0.03a | 1490.67±269.26d | 7.51±0.98c | 0.94±0.05bc |
|  |  |  |  |  |  |  |
| Sample | Fungi | | | | | |
|  | Root | | | Soil | | |
|  | Chao1 | Shannon | Simpson | Chao1 | Shannon | Simpson |
| CP | 168.14±70.16bc | 2.81±1.49a | 0.62±0.26a | 441.64±112.04bc | 5.3±1.06ab | 0.89±0.11ab |
| CT1 | 116.89±7.71bc | 2.34±0.46a | 0.66±0.06a | 505.74±27.18bc | 6.39±0.12a | 0.97±0.00a |
| CT2 | 432.51±249.00a | 4.76±2.24a | 0.82±0.17a | 551.89±39.86b | 5.99±0.3ab | 0.95±0.01ab |
| WP | 166.70±3.30bc | 4.72±0.78a | 0.89±0.05a | 467.30±207.18bc | 4.60±0.42bc | 0.85±0.05ab |
| WT | 285.01±52.16abc | 4.72±0.41a | 0.90±0.01a | 818.80±50.16a | 6.53±1.22a | 0.93±0.08ab |
| WY | 151.74±16.33bc | 2.56±0.39a | 0.60±0.11a | 306.91±12.24cd | 3.39±1.00c | 0.74±0.18b |
| WB | 233.02±42.79abc | 3.98±1.53a | 0.74±0.22a | 405.51±87.67bc | 5.02±1.38abc | 0.87±0.16ab |
| WC | 317.73±248.68ab | 4.63±3.05a | 0.78±0.31a | 578.43±112.30b | 6.45±0.31a | 0.95±0.02a |
| WQ | 80.79±12.84c | 2.45±0.13a | 0.68±0.06a | 154.60±63.74d | 5.16±0.56ab | 0.95±0.01ab |

Note: CP: Cultivated *G. pensilis* samples collected in Pingle County; CT1, CT2: Cultivated *G. pensilis* samples collected in Tiandeng County; WP, WT, WY, WB, WC, WQ: Wild *G. pensilis* samples collected in Pingle County, Tiandeng County, Yanshan District, Binyang County, Cangwu County, and Qintang District. Different letters in the same column indicate signifcant diference at the 0.05 level.

# Supplementary Figures

**
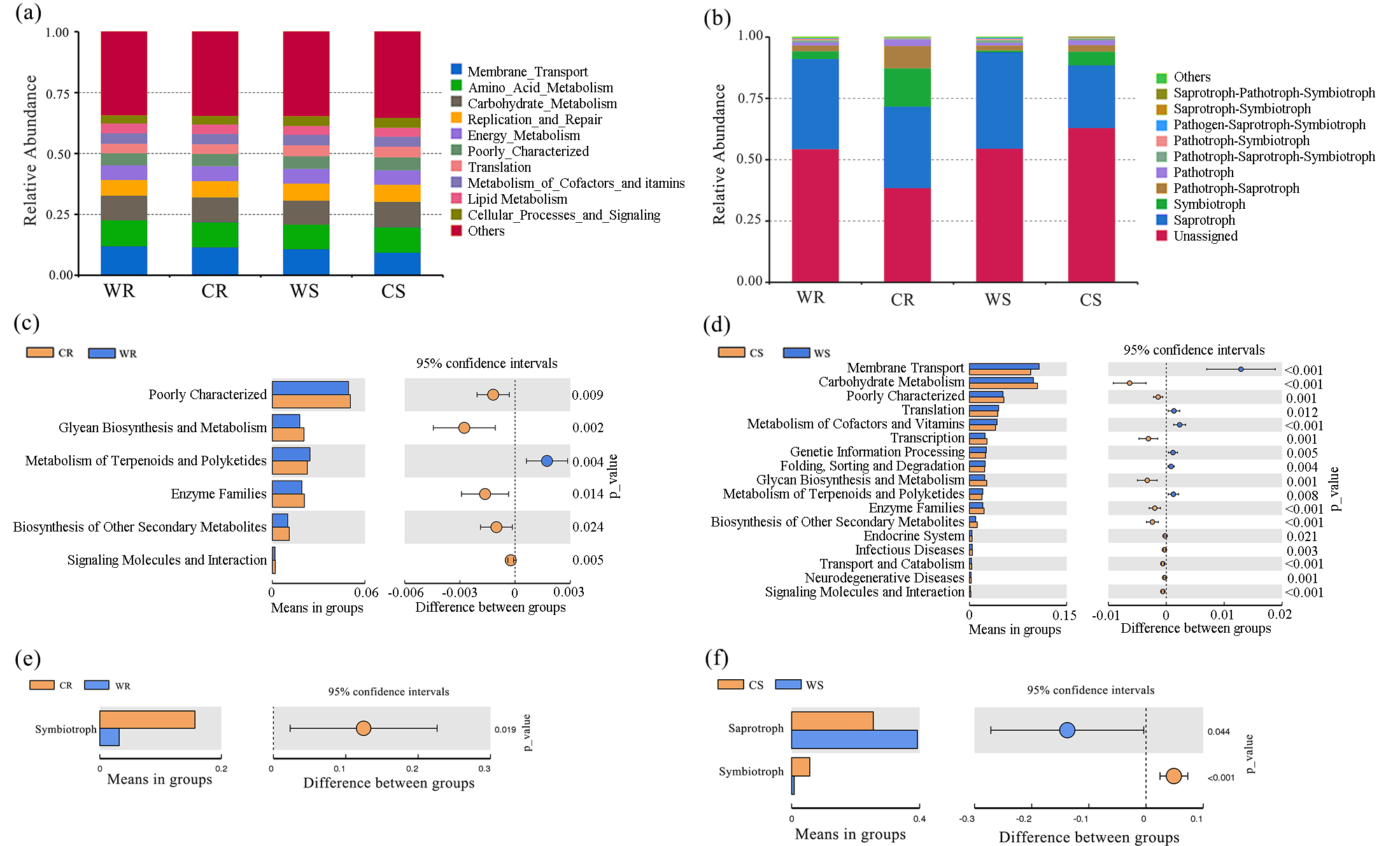
**

**Supplementary Figure 1.** The functional composition and relative abundance histograms of bacterial (a) and fungal (b) communities, as well as the T-test analysis of inter-group functional differences in bacterial (c, d) and fungal (e, f) communities. Inter-group differences are shown with 95% confidence intervals (CR: cultivated *G. pensilis* root; WR: wild *G. pensilis* root; CS: cultivated *G. pensilis* soil; WS: wild *G. pensilis* soil).


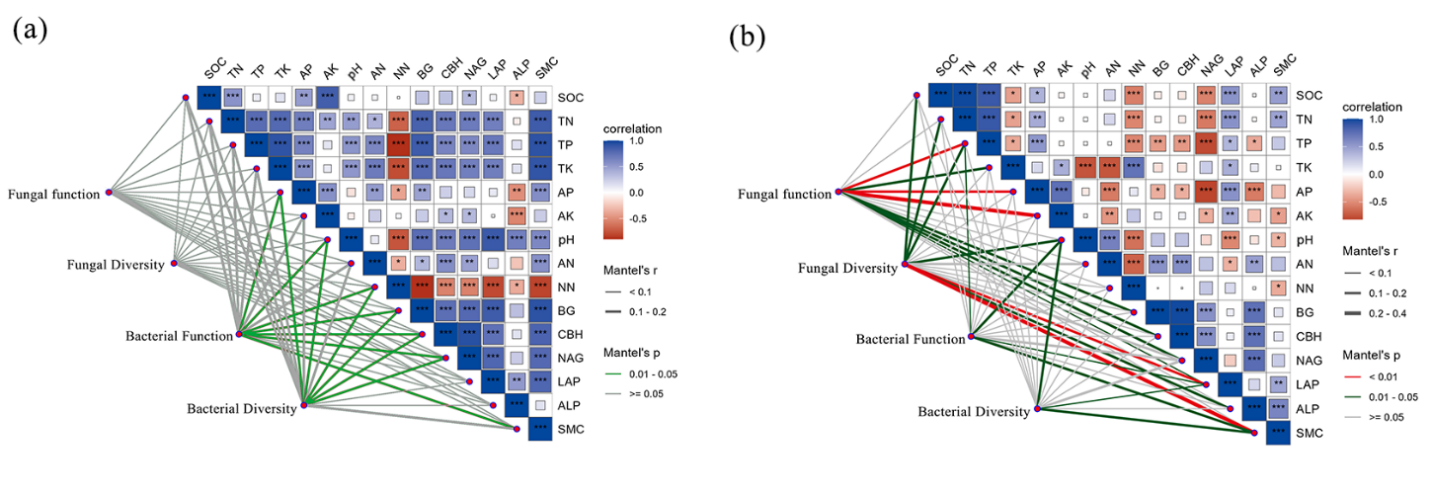
 **Supplementary Figure 2.** The Mantle analysis of environmental factors with microbial communities of cultivated *G. pensilis* (a) and wild *G. pensilis* (b). Line width corresponds to the Mantel's r statistic of the corresponding distance correlation, with edge color indicating statistical significance.
